# Homocysteine enhances the excitability of cultured hippocampal neurons without altering the gene expression of voltage-gated ion channels

**DOI:** 10.1186/s13041-025-01205-x

**Published:** 2025-04-10

**Authors:** Alzbeta Filipova, Matus Tomko, Katarina Ondacova, Lucia Dubiel-Hoppanova, Nikola Chmúrčiaková, Leoš Cmarko, Robin N. Stringer, Norbert Weiss, Lubica Lacinova

**Affiliations:** 1 Center of Biosciences, Institute of Molecular Physiology and Genetics, Slovak Academy of Sciences, Bratislava, Slovakia; 2 Institute of Biology and Medical Genetics, First Faculty of Medicine, Charles University, Prague, Czech Republic; 3 Department of Pathophysiology, Third Faculty of Medicine, Charles University, Prague, Czech Republic

**Keywords:** Hyperhomocysteinemia, Hippocampal excitability, Transcriptomics, Voltage gated ion channels, Intracellular calcium

## Abstract

Elevated plasma homocysteine (Hcy) levels lead to hyperhomocysteinemia, a condition associated with various neurological disorders affecting multiple brain regions, including the hippocampus. In this study, we investigated the effects of exposing cultured rat hippocampal neurons to Hcy concentrations corresponding to mild, moderate, and severe hyperhomocysteinemia. A short 24-hour exposure had minimal effects, whereas prolonged exposure up to 14 days moderately enhanced hippocampal excitability without altering the gene expression of voltage-dependent calcium, sodium, or potassium channels or intracellular calcium levels. These findings suggest that Hcy-induced changes in neuronal excitability may contribute to neuropathologies associated with hyperhomocysteinemia.

**Supplementary Information:**

The online version contains supplementary material available at 10.1186/s13041-025-01205-x.

## Main text

Homocysteine (Hcy) is a non-proteinogenic amino acid produced during the metabolism of methionine to cysteine. Under normal homeostasis, its plasma concentrations ranges between 5 and 14 µM [1]. Alterations in methionine metabolism can lead to elevated Hcy levels, a condition known as hyperhomocysteinemia (HHcy), which is classified as mild (15–30 µM), moderate (31–100 µM), or severe (> 100 µM) [2]. HHcy is associated with a wide range of disorders, including cardiovascular and neurological conditions [2]. Several neurological disorders, such as cognitive impairments [3], seizures, intellectual disabilities, and stroke [4], involve altered hippocampal function. Elevated Hcy levels can influence voltage-dependent ion channels, leading to neuronal hyperexcitability. Indeed, previous studies have shown that exposing primary cultured hippocampal neurons to 100 µM Hcy (severe HHcy) increases their excitability [5]. However, research on the direct effects of Hcy on ion channels remains limited. Here, we performed a detailed analysis of hippocampal neuron excitability across a range of pathophysiological Hcy concentrations.

We assessed passive and active electrophysiological properties of primary cultured neonatal rat hippocampal neurons exposed to acute (24-hour) or chronic (12-day) elevated Hcy concentrations. Acute exposure involved 50, 100, and 300 µM Hcy, while chronic exposure used lower concentrations (30, 50, and 100 µM) to mimic prolonged elevation. Acute exposure primarily affects proteins directly interacting with Hcy, whereas chronic exposure may alter protein expression, as reported for the Ca_V_3.2 T-type calcium channel [6].

**Fig. 1:**
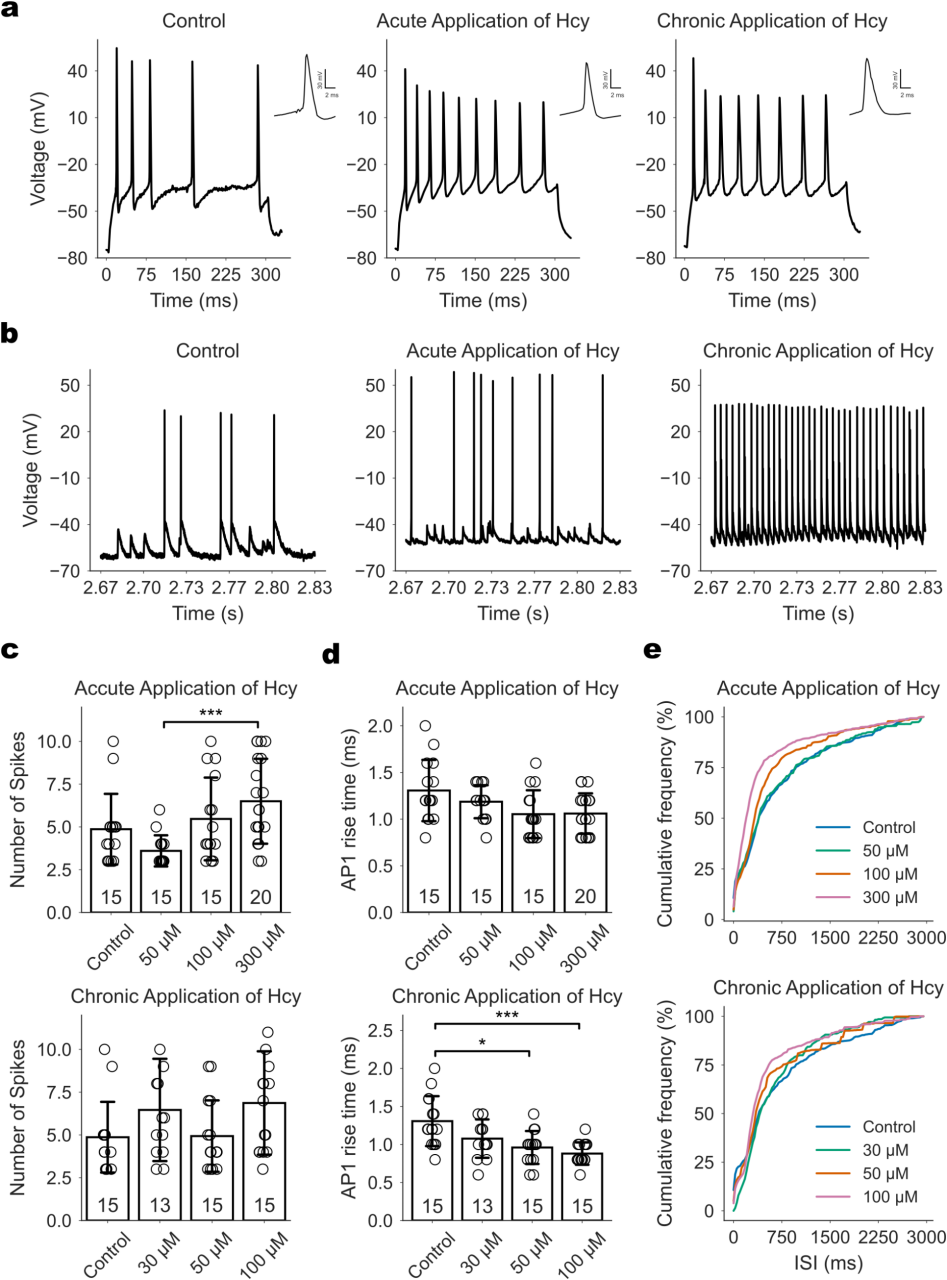
Effect of Hcy on the electrical excitability of cultured hippocampal neurons. **a** Representative recordings of AP series evoked by a 300-ms depolarizing current pulse (300 pA) under control conditions, after acute application of 300 µM Hcy, and after chronic application of 100 µM Hcy, as indicated. Insets show the first AP in each series on an expanded time scale. **b** Representative 160-ms segments extracted from 5-minute recordings of spontaneous neuronal activity under the same conditions as in panel (**a**). **c** Number of APs per series recorded as in panel (**a**) during acute (top panel) and chronic (bottom panel) Hcy application at the indicated concentrations. *p = 0.0119. **d** Rise time of the first AP in each series recorded as in panel (**a**) during acute (top panel) and chronic (bottom panel) Hcy application at indicated concentrations. Numbers within the bars represent the number of neurons analyzed. *p = 0.0176; ***p = 0.0003. **e** Cumulative histograms of interspike intervals (ISI) distributions during 5-minute recordings of spontaneous neuronal activity under control conditions and in the presence of Hcy, as indicated. All experiments were performed on neuronal cultures derived from five independent litters

Resting membrane potential and input resistance remained unchanged across all Hcy concentrations, indicating that passive membrane properties were not affected (Tables S1 and S2). Action potentials (APs) were evoked using 5-ms depolarizing current pulses and analyzed for voltage threshold (V_thresh_), rise time (t_rise_), amplitude (V_ampl_), and half-width (t_halfwidth_). Chronic exposure to 100 µM Hcy accelerated AP rise time and increased AP amplitude, whereas acute exposure had no significant effect on any AP parameter (Tables S1 and S2). To assess AP firing patterns, we induced AP series using 300-ms depolarizing current pulses from a holding potential of -70 mV, selecting a current amplitude that produced regular firing across all Hcy conditions. Acute exposure to 300 µM Hcy mildly increased excitability, as evidenced by a greater number of APs (Fig. 1a and c). In contrast, chronic exposure did not significantly alter the number of APs fired (Fig. 1a and d). The voltage threshold and amplitude of the first AP in a series remained unchanged, while chronic exposure to 50 and 100 µM Hcy accelerated AP rise time (Tables S1 and S2).

As hippocampal neurons matured in culture, they form networks and exhibited spontaneous activity, which we recorded for 5 min without injecting holding current. Cumulative histograms of interspike intervals (ISIs) showed a higher proportion of shorter ISIs following exposure to 100 and 300 µM Hcy in both acute and chronic conditions, indicating increased neuronal excitability (Fig. 1b and e).

Since AP waveforms are shaped by voltage-dependent ion channels (VDICs), we investigated whether prolonged Hcy exposure altered their gene expression. After 14 days of exposure to 30 µM Hcy, a protocol designed to best mimic chronic hyperhomocysteinemia in humans, no significant changes were detected in the expression of voltage-gated calcium, sodium, or potassium channels, their auxiliary subunits, or hyperpolarization-activated cyclic-nucleotide gated channels (Table S3 and Fig S1). Given previous reports that 50 µM Hcy increased intracellular calcium concentration (I_Ca_) via NMDA receptor activation in cortical neurons [7], we also examined whether Hcy exposure influenced I_Ca_ in hippocampal neurons. However, no significant changes in I_Ca_ were observed under any experimental condition (Fig. S2 and Table S2).

Together, our findings demonstrate that both acute and chronic exposure to elevated Hcy concentrations moderately increase hippocampal excitability. Chronic exposure had more pronounced effects, and only concentrations corresponding to moderate to severe HHcy altered neuronal activity. These results are qualitatively consistent with previous findings [5], though quantitative differences may arise from experimental variations. For example, Schaub et al. used embryonic mouse-derived hippocampal cultures recorded between days 9–14 in vitro, whereas our study involved neonatal rat-derived neurons, which may have been at a more advanced stage of maturation. Additionally, species differences in hippocampal neuron excitability have been reported [8]. The absence of VDIC transcriptional changes suggests that the observed excitability increase is more likely due to enhanced excitatory glutamate receptor activity, as reported by other studies [7, 9]. This hypothesis is further supported by our observation that Hcy most prominently increased spontaneous neuronal activity, which is mediated by excitatory synaptic input. However, chronic homocysteine application may still have affected VDIC through downstream mechanisms. For instance, post-translational modifications could have altered channel activity and/or plasma membrane expression, as we previously reported for Ca_v_3.2 T-type calcium channels [6]. Additionally, homocysteine may have influenced the expression of other proteins that regulate VDIC. Altogether, these findings suggest that elevated hippocampal excitability induced by Hcy may play a role in the neuropathological effects of HHcy, including an increased risk of seizures [9, 10], exacerbated ischemic brain damage [9], and cognitive impairment [3, 11].

## Electronic supplementary material

Below is the link to the electronic supplementary material.

**Supplementary Material 1**: **Additional file 1**: Extended methodology and supplemental data.

## Data Availability

All data supporting the findings of this study are available within the paper and its Supplementary Information.

## Abbreviations

Hcy: Homocystein
HHcy: Hyperhomocysteinemia
AP: Action potential
ISI: Interspike interval
V_Thresh_: AP voltage threshold
t_rise_: AP rise time
V_ampl_: AP amplitude
t_halfwidth_: AP half-width
