# Supplementary material for: Homocysteine enhances the excitability of cultured hippocampal neurons without altering the gene expression of voltage-gated ion channels
Source: Mol Brain. 2025 Apr 10;18:31. doi: 10.1186/s13041-025-01205-x (PMC11983933; doi:10.1186/s13041-025-01205-x)
Supplement: Supplementary file 1 — Supplementary Material 1: Additional file 1: Extended methodology and supplemental data. [file 13041_2025_1205_MOESM1_ESM.docx]

Alzbeta Filipova^1^, Matus Tomko^1^, Katarina Ondacova^1^, Lucia Dubiel-Hoppanova^1^, Nikola Chmúrčiaková^2^, Leoš Cmarko^2,3^, Robin N. Stringer^3^, Norbert Weiss^1,3,*^, Lubica Lacinova^1,*^

^1^Center of Biosciences, Institute of Molecular Physiology and Genetics, Slovak Academy of Sciences, Bratislava, Slovakia;

^2^Institute of Biology and Medical Genetics, First Faculty of Medicine, Charles University, Prague, Czech Republic;

^3^Department of Pathophysiology, Third Faculty of Medicine, Charles University, Prague, Czech Republic.

**Additional Methods**

***Animals for electrophysiology***

Female and male Sprague-Dawley rats were obtained from the Department of Toxicology and Laboratory Animals Breeding, Centre of Experimental Medicine of the Slovak Academy of Sciences, Dobra Voda, Slovak Republic. Rats were housed under standard laboratory conditions and fed *ad libitum*. The State Veterinary and Food Administration of the Slovak Republic approved all the experimental procedures under the permit number 6604/2022. The rats were handled according to the Guide for the Care and Use of Laboratory Animals (N.R.C., 1996) and the European Communities Council Directive of September 22, 2010 (2010/63/EU, 74).

***Animals for transcriptomics analysis***

Neonates were obtained from crosses of WHD female and PD/Cub male rats, selected for their high newborn rates and good overall health. The rats were housed in an air-conditioned animal facility and fed *ad libitum*. All animal experiments were conducted in compliance with the Animal Protection Law of the Czech Republic and were approved by the Ethics Committee of the First Faculty of Medicine, Charles University, Prague (Permit Number: MSMT-5938/2022-5).

***Preparation of cell cultures***

Primary cultures of neonatal rat hippocampal neurons were prepared from postnatal day 0 (P0) rats. Hippocampi were microdissected in ice-cold, sterile Hanks' Balanced Salt Solution (HBSS) and incubated in 1 mL of HBSS containing 6 µg/mL papain for 40 minutes at 37°C in a 5% CO_2_ atmosphere. The tissues were then washed with HBSS and gently dissociated by trituration in Neurobasal-A medium supplemented with 10% fetal bovine serum, 1% B-27 supplement, 1% penicillin-streptomycin, and 1% L-glutamine. The cell suspension was centrifuged at 1000 rpm for 5 minutes, and the pellet was resuspended in the same medium. Cells were plated at a density of 5 × 10^4^ cells/mL onto poly-D-lysine-coated petri dishes. On the second day, half of the medium was replaced with Neurobasal-A medium containing 1% B-27, 1% penicillin-streptomycin, 1% L-glutamine, and arachidonic acid. Half of the medium was subsequently refreshed every second day for 14 days.

***Application of Homocysteine***

For transcriptomics analysis, half of the culture dishes were treated with 30 µM Hcy, while the remaining half served as untreated control.

Electrophysiological measurements were performed on days 12-14 *in vitro*. Acute Hcy application (50 µM, 100 µM, and 300 µM) was performed 24 hours before the measurement. Chronic Hcy treatment (30 µM, 50 µM, and 100 µM) was initiated on day 1 *in vitro* and maintained throughout the culture period. One-quarter of the dishes served as untreated control, while remaining three-quarters were exposed with Hcy at indicated concentrations.

***Patch clamp electrophysiology***

Whole-cell current-clamp configuration of the patch-clamp technique was used in all experiments. Signals were recorded using a HEKA EPC10 amplifier (HEKA Electronics, Lambrecht, Germany) controlled by Patchmaster v2x73.3 (HEKA Electronics, Lambrecht/Pfalz, Germany) software.

The extracellular solution for primary hippocampal cultures contained (in mM): 130 NaCl; 3 KCl; 10 HEPES; 2 CaCl_2_; 1 MgCl_2_; 10 D-glucose; pH was adjusted to 7.4 with NaOH. The intracellular solution contained (in mM): 120 K-gluconate; 20 KCl; 2 MgCl_2_; 2 Na_2_ATP; 0.25 Na_2_GTP; 10 HEPES; pH was adjusted to 7.4 with KOH (all chemicals from Sigma‒Aldrich, Germany).

The resting membrane potential (V_rest_) of each neuron was measured immediately after establishing the whole-cell configuration and switching to current-clamp mode. During all subsequent experiments, the membrane potential was held at -70 mV. Input resistance (R_inp_) was determined using a series of 250 ms hyperpolarizing current pulses, with stepwise amplitude decreases of -25 pA. The membrane potential was recorded at the end plateau of each current pulse, and R_inp_ was calculated from the voltage‒current relationship using Ohm’s law. Single action potentials (APs) were evoked by 5 ms depolarizing current pulses with stepwise amplitude increases of +50 pA. AP series were evoked using 300 ms depolarizing current pulses with +50 pA amplitude increments. Spontaneous neuronal activity was recorded for 5 minutes at each neuron’s native membrane potential.

Experimental data were analysed using Fitmaster v2x73.3 (HEKA Electronics, Lambrecht/Pfalz, Germany) and Origin 2022 Academic (OriginLab Co., Northampton, MA, USA) software. Prior to feature extraction, voltage traces were filtered using the Savitzky-Golay filter (scipy.signal.savgol_filter) [1], applying a 5 ms window for sharp voltage changes (e.g., APs) and a 15 ms window for other segments. Electrophysiological features were extracted from individual traces using the open-source Electrophysiological Feature Extraction Library (eFEL) [2] combined with a custom Python script. Default eFEL settings were modified to optimize spike detection and AP threshold calculations: eFEL.Threshold=0.0 mV; eFEL.inter_step=0.05 ms for single APs and 0.2 ms for AP series; eFEL.DerivativeWindow=3.0 ms; eFEL.DerivativeThreshold=5.0 mV for AP series and 10.0 mV for single APs (or higher if threshold detection was suboptimal). If noise led to incorrect spike detection, manual corrections were applied, and the affected features were recalculated.

***Intracellular calcium concentration (I_Ca_)***

For I_Ca_ measurement, 1x Hank´s Balanced Salt Solution (HBSS) was used, containing (in mM): 137 NaCl; 5.4 KCl; 0.5 MgCl_2_ x 6H2O; 0.4 MgSO_4_x7H_2_O; 0.44 KH_2_PO_4_; 0.34 Na_2_HPO_4_ x 7H_2_O; 1.3 CaCl_2_; 4.2 NaHCO_3_; 5.5 D glucose). Cells were first washed three times with 1x HBSS containing 1mg/ml BSA. After washing, Fura2-AM (2mM, Sigma Aldrich 47989) was prepared in 1x HBSS + BSA at final concentration of 4 nM/ml and applied to the cells, followed by incubation for 1 hour in 37°C in 5% CO_2_. After incubation, cells were washed three times in 1x HBSS without BSA and incubated in 1x HBSS for 30 minutes in 37°C in 5% CO_2_ before measurement. I_Ca_ was measured using a Synergy H4 Multi-Mode Microplate Reader (BioTek, USA) with excitation wavelengths of 340 nm and 380 nm, and an emission wavelength 510 nm. Four wells per experimental group were analysed. All experiments were conducted on Day 14 *in vitro*.

***Transcriptomic analysis***

Total RNA was isolated from cultured neonatal rat hippocampal neurons using TRIzol^TM^ Reagent (Invitrogen) according to the manufacturer’s instructions. RNA quality and quantification were assessed using a BioPhotometer (Eppendorf) and a 2100 Bioanalyzer system (Agilent Technologies). The isolated total RNA was used to prepare cDNA using SuperScript IV reverse transcriptase (Thermo Fisher Scientific) according to manufacturer instructions. Quantitative real-time-PCR (qPCR) was performed using TaqMan® Fast Universal PCR Master Mix (Thermo Fisher Scientific) and a 7900HT thermocycler (Applied Biosystems). Custom TaqMan™ Array Plates (Thermo Fisher Scientific) were used to investigate the effect of homocysteine on the gene expression of different voltage-gated ion channels. The 96-well plate contained 92 assays of genes encoding calcium, sodium, and potassium channels, along with 4 reference gene assays (18S, GAPDH, HPRT1, GUSB) as endogenous controls (Supplementary Table 2). For qPCR analysis, cycle threshold (Ct) values of target genes were normalized to beta-glucuronidase (GUSB), which served as the internal control. All experiments were performed in triplicates, and relative gene expression was quantified using the ΔΔCt method.

***Statistical analysis***

All results are presented means ± S.D. Statistical analyses were performed using the SciPy [1], statsmodels [2], and scikit_posthocs [3] Python packages. Data normality was assessed using the D’Agostino and Pearson’s test (scipy.stats.normaltest) when the number of datapoints exceeded 20; otherwise, the Shapiro-Wilk test (scipy.stats.shapiro) was used. For normally distributed data, a one-way ANOVA (statsmodels.stats.anova) was applied, followed by Tukey’s HSD (statsmodels.stats.multicomp) post-hoc test to pairwise comparisons. For non-normally distributed data, the Kruskal-Wallis H-test (scipy.stats.kruskal) was used, followed by Dunn’s post-hoc test (scikit_posthocs) with Bonferroni correction for pairwise comparisons.

**Supplemental Table 1** Summary of passive electrical properties, single AP parameters, and first AP parameters in a series evoked by a depolarizing current pulse. The number of neurons analysed is indicated in brackets. Neurons were derived from five independent litters.

| Parameter | | Control | Acute application of Hcy | | | Chronic application of Hcy | | |
| --- | --- | --- | --- | --- | --- | --- | --- | --- |
|  |  |  | 50 µM | 100 µM | 300 µM | 30 µM | 50 µM | 100 µM |
| V_rest_ (mV) | | -68.7 ± 5.1 (21) | -68.9 ± 4.6 (23) | -68.0 ± 4.8 (23) | -70.3 ± 5.3 (26) | -68.1 ± 4.9 (26) | -70.6 ± 6.2 (22) | -68.6 ± 4.1 (25) |
| R_inp_ (MΩ) | | 283.3±121.4 (21) | 312.7±137.8 (23) | 317.9±120.0 (22) | 342.4±96.6 (26) | 240.5±85.4 (26) | 259.2±127.9 (22) | 225.9±105.4 (25) |
| Single AP | t_rise_ (ms) | 0.9±0.2 (20) | 0.9±0.2 (22) | 0.9±0.2 (22) | 0.9±0.2 (26) | 0.9±0.2 (26) | 0.9±0.1 (22) | 0.8±0.1 (25) |
|  | V_ampl_ (mV) | 73.7±10.2 (20) | 74.8±8.6 (22) | 74.4±11.0 (22) | 81.5±10.5 (26) | 80.7±10.3 (26) | 77.9±8.8 (22) | 86.3±12.1 (25) |
|  | t_halfwidth_ (ms) | 1.3±0.2 (20) | 1.3±0.4 (22) | 1.2±0.2 (22) | 1.3±0.2 (26) | 1.4±0.3 (26) | 1.4±0.3 (22) | 1.1±0.3 (25) |
|  | V_thresh_ (mV) | -27.8±4.0 (20) | -28.2±3.7 (22) | -28.3±3.6 (22) | -29.9±5.1 (26) | -30.3±4.6 (26) | -27.5±3.9 (22) | -30.2±5.8 (25) |
| AP series | V_thresh_ (mV) | -34.3±3.5 (15) | -32.7±3.5 (15) | -31.8±3.8 (15) | -33.5±2.7 (20) | -34.2±2.6 (13) | -31.0±4.1 (15) | -32.8±3.5 (15) |
|  | V_ampl_ (mV) | 80.3±8.4 (15) | 77.8±9.0 (15) | 79.8±10.6 (15) | 85.0±9.0 (20) | 85.9±7.5 (13) | 83.3±10.2 (15) | 87.5±10.3 (15) |

**Supplemental Table 2** Summary of statistical comparisons of electrophysiological parameters relative to the control group. Additionally. significant differences were found between: 30 µM (chronic Hcy application) and 100 µM (chronic Hcy application) for single AP rise time (t_rise_, p = 0.0119); 50 µM (chronic Hcy application) and 100 µM (chronic Hcy application) for the single AP amplitude (V_ampl_, p = 0.0357); and 50 µM (acute Hcy application) and 300 µM (acute Hcy application) for AP series spike count (p = 0.00043). n.s., non-significant.

| Parameter | | Control | Acute application of Hcy | | | Chronic application of Hcy | | |
| --- | --- | --- | --- | --- | --- | --- | --- | --- |
|  |  |  | 50 µM | 100 µM | 300 µM | 30 µM | 50 µM | 100 µM |
| V_rest_ | |  | n. s. | n. s. | n. s. | n. s. | n. s. | n. s. |
| R_inp_ | |  | n. s. | n. s. | n. s. | n. s. | n. s. | n. s. |
| Single AP | t_rise_ |  | n. s. | n. s. | n. s. | n. s. | n. s. | 0.0147 |
|  | V_ampl_ |  | n. s. | n. s. | n. s. | n. s. | n. s. | 0.0007 |
|  | t_halfwidth_ |  | n. s. | n. s. | n. s. | n. s. | n. s. | n. s. |
|  | V_thresh_ |  | n. s. | n. s. | n. s. | n. s. | n. s. | n. s. |
| AP series | V_thresh_ |  | n. s. | n. s. | n. s. | n. s. | n. s. | n. s. |
|  | V_ampl_ |  | n. s. | n. s. | n. s. | n. s. | n. s. | n. s. |
|  | t_rise_ |  | n. s. | n. s. | n. s. | n. s. | 0.0176 | 0.0003 |
|  | Spikecount |  | n. s. | n. s. | n. s. | n. s. | n. s. | n. s. |
| I_Ca_ | |  | n. s. | n. s. | n. s. | n. s. | n. s. | n. s. |

**Supplemental Table 3.** Array target genes and their corresponding assay IDs.

| **#** | **Assay ID** | **Gene Symbol** | **Gene** |
| --- | --- | --- | --- |
| **Reference genes** | | | |
| **1** | Hs99999901_s1 | 18S | eukaryotic 18S rRNA |
| **2** | Rn99999916_s1 | GAPDH | glyceraldehyde-3-phosphate dehydrogenase |
| **3** | Rn01527840_m1 | HPRT1 | hypoxanthine phosphoribosyltransferase 1 |
| **4** | Rn00566655_m1 | GUSB | glucuronidase. beta |
| **Calcium channels** | | | |
| **5** | Rn01512403_m1 | CACNA1A | calcium channel. voltage-dependent. P/Q type. alpha 1A subunit |
| **6** | Rn00595911_m1 | CACNA1B | calcium channel. voltage-dependent. N type. alpha 1B subunit |
| **7** | Rn00568820_m1 | CACNA1D | calcium channel. voltage-dependent. L type. alpha 1D subunit |
| **8** | Rn00494444_m1 | CACNA1E | calcium channel. voltage-dependent. R type. alpha 1E subunit |
| **9** | Rn00586734_m1 | CACNA1F | calcium channel. voltage-dependent. L type. alpha 1F subunit |
| **10** | Rn00581051_m1 | CACNA1G | calcium channel. voltage-dependent. T type. alpha 1G subunit |
| **11** | Rn01460348_m1 | CACNA1H | calcium channel. voltage-dependent. T type. alpha 1H subunit |
| **12** | Rn01505208_m1 | CACNA1I | calcium channel. voltage-dependent. T type. alpha 1I subunit |
| **13** | Rn00569267_m1 | CACNB1 | calcium channel. voltage-dependent. beta 1 subunit |
| **14** | Rn00587789_m1 | CACNB2 | calcium channel. voltage-dependent. beta 2 subunit |
| **15** | Rn00432233_m1 | CACNB3 | calcium channel. voltage-dependent. beta 3 subunit |
| **16** | Rn01449787_m1 | CACNB4 | calcium channel. voltage-dependent. beta 4 subunit |
| **17** | Rn00584355_m1 | CACNG2 | calcium channel. voltage-dependent. gamma subunit 2 |
| **18** | Rn00589900_m1 | CACNG3 | calcium channel. voltage-dependent. gamma subunit 3 |
| **19** | Rn00589903_m1 | CACNG4 | calcium channel. voltage-dependent. gamma subunit 4 |
| **20** | Rn00589905_m1 | CACNG5 | calcium channel. voltage-dependent. gamma subunit 5 |
| **21** | Rn00519216_m1 | CACNG7 | calcium channel. voltage-dependent. gamma subunit 7 |
| **Potassium channels** | | | |
| **22** | Rn00670384_m1 | HCN1 | hyperpolarization-activated cyclic nucleotide-gated potassium channel |
| **23** | Rn01408572_mH | HCN2 | hyperpolarization activated cyclic nucleotide-gated potassium channel 2 |
| **24** | Rn00597355_s1 | KCNA1 | potassium voltage-gated channel. shaker-related subfamily. member 1 |
| **25** | Rn02769834_s1 | KCNA2 | potassium channel. voltage gated shaker related subfamily A. member 2 |
| **26** | Rn00570552_s1 | KCNA3 | potassium channel. voltage gated shaker related subfamily A. member 3 |
| **27** | Rn00564245_s1 | KCNA5 | potassium channel. voltage gated shaker related subfamily A. member 5 |
| **28** | Rn01492950_s1 | KCNA6 | potassium channel. voltage gated shaker related subfamily A. member 6 |
| **29** | Rn01476090_m1 | KCNA7 | potassium channel. voltage gated shaker related subfamily A. member 7 |
| **30** | Rn00568877_m1 | KCNAB1 | potassium channel. voltage-gated shaker related subfamily A regulatory beta subunit 1 |
| **31** | Rn00568891_m1 | KCNAB2 | potassium channel. voltage-gated shaker-related subfamily A regulatory beta subunit 2 |
| **32** | Rn00755102_m1 | KCNB1 | potassium channel. voltage gated Shab-related subfamily B. member 1 |
| **33** | Rn07359695_m1 | KCNB2 | potassium channel. voltage gated Shab-related subfamily B. member 2 |
| **34** | Rn00563433_m1 | KCNC1 | potassium channel. voltage gated Shaw-related subfamily C. member 1 |
| **35** | Rn01748431_m1 | KCNC4 | potassium channel. voltage gated Shaw-related subfamily C. member 4 |
| **36** | Rn01525167_m1 | KCND1 | potassium channel. voltage gated Shal-related subfamily D. member 1 |
| **37** | Rn00581941_m1 | KCND2 | potassium channel. voltage-gated Shal-related subfamily D. member 2 |
| **38** | Rn01534234_m1 | KCND3 | potassium channel. voltage-gated Shal-related subfamily D. member 3 |
| **39** | Rn02094595_s1 | KCNE1 | potassium channel. voltage-gated Isk-related subfamily E regulatory beta subunit 1 |
| **40** | Rn02094913_s1 | KCNE2 | potassium channel. voltage-gated Isk-related subfamily E regulatory beta subunit 2 |
| **41** | Rn04176290_s1 | KCNF1 | potassium channel. voltage-gated modifier subfamily F. member 1 |
| **42** | Rn01750192_m1 | KCNG1 | potassium channel. voltage-gated modifier subfamily G. member 1 |
| **43** | Rn01399698_m1 | KCNG2 | potassium channel. voltage gated modifier subfamily G. member 2 |
| **44** | Rn00695201_m1 | KCNG3 | potassium channel. voltage gated modifier subfamily G. member 3 |
| **45** | Rn01432541_m1 | KCNG4 | potassium channel. voltage gated modifier subfamily G. member 4 |
| **46** | Rn00582012_m1 | KCNH1 | potassium channel. voltage gated eag related subfamily H. member 1 |
| **47** | Rn00588515_m1 | KCNH2 | potassium channel. voltage gated eag related subfamily H. member 2 |
| **48** | Rn00567367_m1 | KCNH3 | potassium channel. voltage gated eag related subfamily H. member 3 |
| **49** | Rn00586212_m1 | KCNH4 | potassium channel. voltage gated eag related subfamily H. member 4 |
| **50** | Rn00592402_m1 | KCNH5 | potassium channel. voltage gated eag related subfamily H. member 5 |
| **51** | Rn00672436_m1 | KCNH6 | potassium channel. voltage gated eag related subfamily H. member 6 |
| **52** | Rn00590940_m1 | KCNH7 | potassium channel. voltage gated eag related subfamily H. member 7 |
| **53** | Rn00566732_m1 | KCNJ1 | potassium channel. inwardly rectifying subfamily J. member 1 |
| **54** | Rn01764077_s1 | KCNJ11 | potassium channel. inwardly rectifying subfamily J. member 11 |
| **55** | Rn02533449_s1 | KCNJ12 | potassium channel. inwardly rectifying subfamily J. member 12 |
| **56** | Rn00586020_m1 | KCNJ13 | potassium channel. inwardly rectifying subfamily J. member 13 |
| **57** | Rn00821873_m1 | KCNJ14 | potassium channel. inwardly rectifying subfamily J. member 14 |
| **58** | Rn00434617_m1 | KCNJ3 | potassium channel. inwardly rectifying subfamily J. member 3 |
| **59** | Rn01502359_m1 | KCNJ4 | potassium channel. inwardly rectifying subfamily J. member 4 |
| **60** | Rn00572452_m1 | KCNK1 | potassium channel. two pore domain subfamily K. member 1 |
| **61** | Rn00576558_m1 | KCNK10 | potassium channel. two pore domain subfamily K. member 10 |
| **62** | Rn02132664_s1 | KCNK12 | potassium channel. two pore domain subfamily K. member 12 |
| **63** | Rn00755451_m1 | KCNK13 | potassium channel. two pore domain subfamily K. member 13 |
| **64** | Rn02349659_m1 | KCNK15 | potassium channel. two pore domain subfamily K. member 15 |
| **65** | Rn01537127_m1 | KCNK16 | potassium channel. two pore domain subfamily K. member 16 |
| **66** | Rn00570571_m1 | KCNMB1 | calcium-activated potassium channel subunit beta-1 |
| **67** | Rn00597042_m1 | KCNK2 | potassium channel. two pore domain subfamily K. member 2 |
| **68** | Rn04223042_m1 | KCNK3 | potassium channel. two pore domain subfamily K. member 3 |
| **69** | Rn00587450_m1 | KCNK4 | potassium channel. two pore domain subfamily K. member 4 |
| **70** | Rn01755927_m1 | KCNK5 | potassium channel. two pore domain subfamily K. member 5 |
| **71** | Rn00821542_g1 | KCNK6 | potassium channel. two pore domain subfamily K. member 6 |
| **72** | Rn01498282_m1 | KCNK7 | potassium channel. two pore domain subfamily K. member 7 |
| **73** | Rn00755967_m1 | KCNK9 | potassium channel. two pore domain subfamily K. member 9 |
| **74** | Rn00582881_m1 | KCNMA1 | potassium channel. calcium activated large conductance subfamily M alpha. member 1 |
| **75** | Rn00576373_m1 | KCNN4 | potassium channel. calcium activated intermediate/small conductance subfamily N alpha. member 4 |
| **76** | Rn00583376_m1 | KCNQ1 | potassium channel. voltage-gated KQT-like subfamily Q. member 1 |
| **77** | Rn00591249_m1 | KCNQ2 | potassium channel. voltage-gated KQT-like subfamily Q. member 2 |
| **78** | Rn00580995_m1 | KCNQ3 | potassium channel. voltage-gated KQT-like subfamily Q. member 3 |
| **79** | Rn01518851_m1 | KCNQ4 | potassium channel. voltage-gated KQT-like subfamily Q. member 4 |
| **80** | Rn01512013_m1 | KCNQ5 | potassium channel. voltage-gated KQT-like subfamily Q. member 5 |
| **81** | Rn00588597_m1 | KCNS1 | potassium voltage-gated channel. modifier subfamily S. member 1 |
| **82** | Rn01772109_m1 | KCNV2 | potassium channel. voltage-gated modifier subfamily V. member 2 |
| **Sodium channels** | | | |
| **83** | Rn00568393_m1 | SCN10A | sodium channel. voltage-gated. type X. alpha subunit |
| **84** | Rn00570487_m1 | SCN11A | sodium channel. voltage-gated. type XI. alpha subunit |
| **85** | Rn00578439_m1 | SCN1A | sodium channel. voltage-gated. type I. alpha subunit |
| **86** | Rn00441210_m1 | SCN1B | sodium channel. voltage-gated. type I. beta subunit |
| **87** | Rn00680558_m1 | SCN2A | sodium channel. voltage-gated. type II. alpha subunit |
| **88** | Rn00563554_m1 | SCN2B | sodium channel. voltage-gated. type II. beta subunit |
| **89** | Rn01485335_m1 | SCN3A | sodium channel. voltage-gated. type III. alpha subunit |
| **90** | Rn01422019_m1 | SCN3B | sodium channel. voltage-gated. type III. beta subunit |
| **91** | Rn01461132_m1 | SCN4A | sodium channel. voltage-gated. type IV. alpha subunit |
| **92** | Rn01418017_m1 | SCN4B | sodium channel. voltage-gated. type IV. beta subunit |
| **93** | Rn00565502_m1 | SCN5A | sodium channel. voltage-gated. type V. alpha subunit |
| **94** | Rn00581647_m1 | SCN7A | sodium channel. voltage-gated. type VII. alpha subunit |
| **95** | Rn00570506_m1 | SCN8A | sodium channel. voltage gated. type VIII. alpha subunit |
| **96** | Rn00591020_m1 | SCN9A | sodium channel. voltage-gated. type IX. alpha subunit |


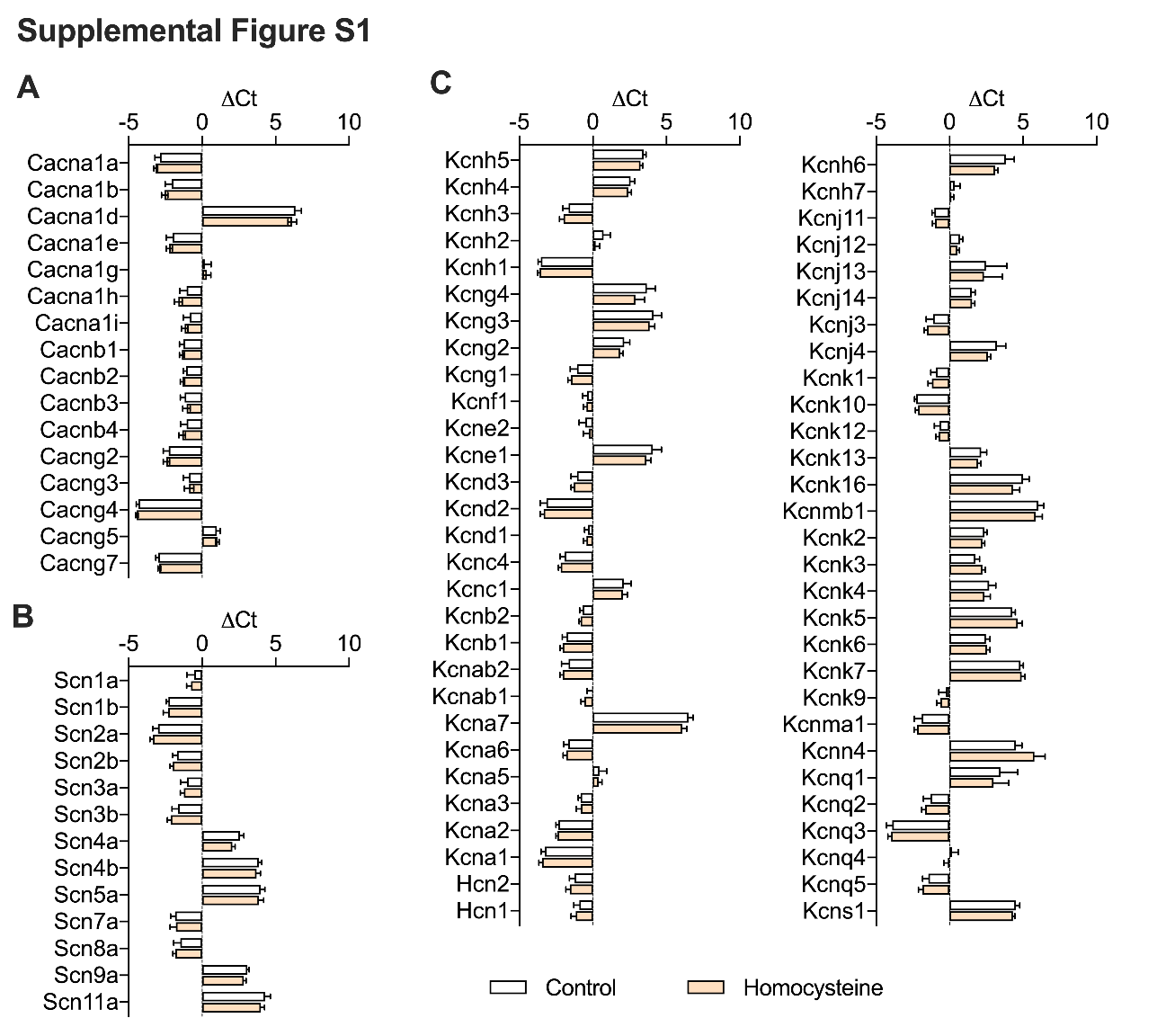


**Fig. S1** Effect of chronic Hcy exposure on the transcription of genes encoding principal and auxiliary subunits of voltage-gated calcium, sodium, and potassium channels in cultured hippocampal neurons (listed in the **Supplementary Table 2**). No significant changes were detected in any of these transcripts.

**Supplemental Figure S2.**


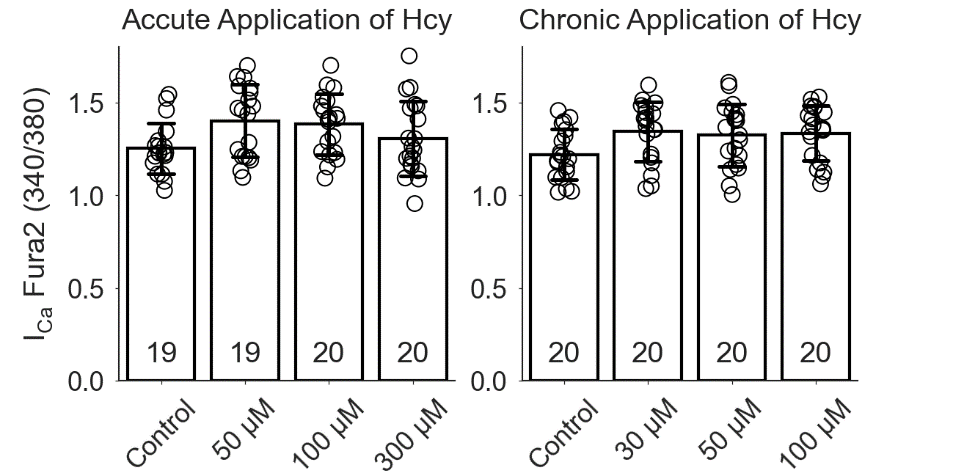


**Fig. S2** Effect of acute and chronic Hcy exposure on free intracellular calcium concentration in cultured hippocampal neurons. No significant changes were observed under any condition.

**References**

1. Virtanen P, Gommers R, Oliphant TE, Haberland M, Reddy T, Cournapeau D, et al. SciPy 1.0: fundamental algorithms for scientific computing in Python. Nat Methods [Internet]. 2020 [cited 2021 May 18];17:261–72. Available from: https://doi.org/10.1038/s41592-019-0686-2

2. Seabold S, Perktold J. Statsmodels: Econometric and Statistical Modeling with Python. 9th Python in Science Conference [Internet]. Austin; 2010. p. 92–6. Available from: https://doi.curvenote.com/10.25080/Majora-92bf1922-011

3. Terpilowski M. scikit-posthocs: Pairwise multiple comparison tests in Python. J Open Source Softw. 2019;4:1169.
